# Formation of nanoripples on ZnO flat substrates and nanorods by gas cluster ion bombardment

**DOI:** 10.3762/bjnano.11.29

**Published:** 2020-02-24

**Authors:** Xiaomei Zeng, Vasiliy Pelenovich, Bin Xing, Rakhim Rakhimov, Wenbin Zuo, Alexander Tolstogouzov, Chuansheng Liu, Dejun Fu, Xiangheng Xiao

**Affiliations:** 1 Department of Physics and Key Laboratory of Artificial Micro- and Nano-structures of Ministry of Education, Hubei Nuclear Solid Physics Key Laboratory and Center for Ion Beam Application, School of Physics and Technology, Wuhan University, Wuhan 430072, China; 2 School of Power & Mechanical Engineering, Wuhan University, Wuhan 430072, China; 3 Ryazan State Radio Engineering University, Gagarin Str. 59/1, Ryazan 390005, Russian Federation; 4 Centre for Physics and Technological Research (CeFITec), Dept. de Física da Faculdade de Ciências e Tecnologia (FCT), Universidade Nova de Lisboa, Caparica 2829-516, Portugal; 5 Shenzhen Institute of Wuhan University

**Keywords:** cluster ion bombardment, gas cluster ion beam, surface ripples, ZnO nanorods

## Abstract

In the present study Ar^+^ cluster ions accelerated by voltages in the range of 5–10 kV are used to irradiate single crystal ZnO substrates and nanorods to fabricate self-assembled surface nanoripple arrays. The ripple formation is observed when the incidence angle of the cluster beam is in the range of 30–70°. The influence of incidence angle, accelerating voltage, and fluence on the ripple formation is studied. Wavelength and height of the nanoripples increase with increasing accelerating voltage and fluence for both targets. The nanoripples formed on the flat substrates remind of aeolian sand ripples. The ripples formed at high ion fluences on the nanorod facets resemble well-ordered parallel steps or ribs. The more ordered ripple formation on nanorods can be associated with the confinement of the nanorod facets in comparison with the quasi-infinite surface of the flat substrates.

## Introduction

The formation of self-assembled nanoscale surface structures using off-normal ion irradiation has a few advantages over traditional photolithography techniques, i.e., the absence of fundamental restrictions for the size reduction of the formed structures and cost-effective production. However, the formation of self-assembled structures still suffers from poor control as well as the lack of understanding regarding the mechanisms involved [1]. Nowadays, self-assembled surface nanoscale structures are of interest in many applications. Substrates with nanoscale ripples are excellent templates for the deposition of semiconductor quantum dots [2]. Arrays of metallic nanoparticles or nanowires aligned on dielectric surfaces with nanoripples are ideal for research on plasmonics [3]. Ag nanoparticle arrays created on rippled silicon surfaces have demonstrated excellent sensing of molecules through surface-enhanced Raman spectroscopy [4]. Ion beam formation of nanoscale ripples has emerged as a versatile method to imprint uniaxial magnetic anisotropy [5] and to control the magnetic texture of thin films [6]. Formation of self-assembled surface nanoripple structures by monoatomic off-normal ion irradiation was discovered by Navez et al. [7] and studied in details by Carter and co-workers [8]. Later, a theoretical explanation of the ripple formation was given by Bradley and Harper (BH) and Bradley and Shipman (BS) using Sigmund’s sputtering theory [9–10]. These models consider both erosion of the surface by the ion beam and thermal diffusion of the target atoms.

Recently, the gas cluster ion beams (GCIB) technique has been introduced as a mask-free method to produce nanostructures on solid surfaces [11–12]. Among its advantages are the lack of chemical contamination and the low damage to the subsurface layer. A gas cluster is a relatively stable particle, which consists of about a thousand atoms or molecules bound by van der Waals interactions. Cluster beams have attracted great attention because of their unusual properties, such as, the high mass–charge ratio, the ability to transport more material than atomic beams, the high energy density when a cluster interacts with a surface, and nonlinear sputtering effects [13]. One of the most prominent properties of cluster beams is the smoothing effect on moderately rough surfaces [14]. The smoothing effect takes place at normal incidence of the cluster beam upon the surface plane, while at off-normal incidence nanometer-sized ripples are formed on the substrate surface. When an energetic cluster collides with the surface temperature and pressure in the impact zone increase sharply due to the high energy density, which results in melting, partial vaporization and ejection of the target material as well as the formation of nanometer-sized craters. If the GCIB incidence is normal to the surface the formed craters have circular symmetrical rims, while at off-normal incidence the crater rims become asymmetrical and most of the ejected substrate material is deposited downstream nearby the impact spot at a distance of about 10 nm [15]. This small impact accumulation gives rise to the formation of ripples with increasing cluster fluence.

Toyoda et al. have studied the influence of incidence angle and cluster size on the ripple formation on Au surfaces. The most effective ripple growth was found for incidence angles in the range of 45–60° [11]. The angle and ion fluence dependencies of ripples formed on single crystal Si surfaces were studied by Lozano and co-workers [12]. The ripples on the Si surface are quite similar to those formed on gold substrates, suggesting a small influence of the sort of material or the crystal orientation of the substrate on the ripple formation. Recently, Saleem et al. have proposed to use the nanoripple structures formed by GCIB for plasmonic and biomolecular sensing applications [16–17].

In all above-mentioned studies planar substrates have been employed, such as Si wafers, bulk Au samples or SiO_2_ films. There are very few papers on the ripple formation on the surface of confined nanostructures both by monoatomic and cluster ion irradiation. Therefore, in this research we study features of nanoripple formation on the facets of ZnO nanorods irradiated by GCIB. The modification effects of the gas cluster ion beams on the nanostructured targets at different incidence angles, accelerating voltages, and ion fluences are studied. The differences between nanoripple formation on planar ZnO substrates and on nanorods are also discussed. The results obtained in this study are of interest in the application of ZnO nanostructures for, e.g., gas sensing, solar cells, or field emitters, where controlled surface morphologies are required.

## Experimental

We have grown ZnO nanorods on Si(100) substrates (HF-Kejing Materials Technology Co., Ltd.) by means of a two-step hydrothermal (HT) growth process. The first step is the synthesis of a seed layer and the second step is the ZnO nanorod growth. To prepare the seed layer solution we stirred 5 mM Zn(CH_3_COO)_2_·2H_2_O (zinc acetate dehydrate, 99.9%, Sinopharm Chemical Reagent Co., Ltd) in 10 mL of deionized (DI) water for 4 h at 60 °C. Then, the solution was heated in an oven for 20 h at 60 °C. Next, ZnO seeds were synthesized on the Si substrates through spin-coating. First, a drop of the seed solution was put onto the cleaned substrate at 700 rpm for 10 s and then at 2000 rpm for 60 s to coat the substrate. Next, the coated substrates were put on a heating plate for 10 min at 170 °C in air. These coating procedures were repeated three times. Finally, the coated substrates were heated at 350 °C for 20 min in air to obtain layer of crystalline ZnO islets. The solution for HT growth of the nanorods was prepared using 50 mM Zn(CH_3_COO)_2_·2H_2_O and 25 mM hexamethylenetetramine (99.9%, Sinopharm Chemical Reagent Co., Ltd) in 25 mL of DI water. The nanorods were grown on the Si substrates in a Teflon reactor for a period of 4 h at 95 °C. To compare the ripples formation on the nanorods and flat surfaces, we also used polished ZnO(0001) substrates (10 × 10 × 0.5 mm, HF-Kejing Materials Technology Co., Ltd.).

**Figure 1:**
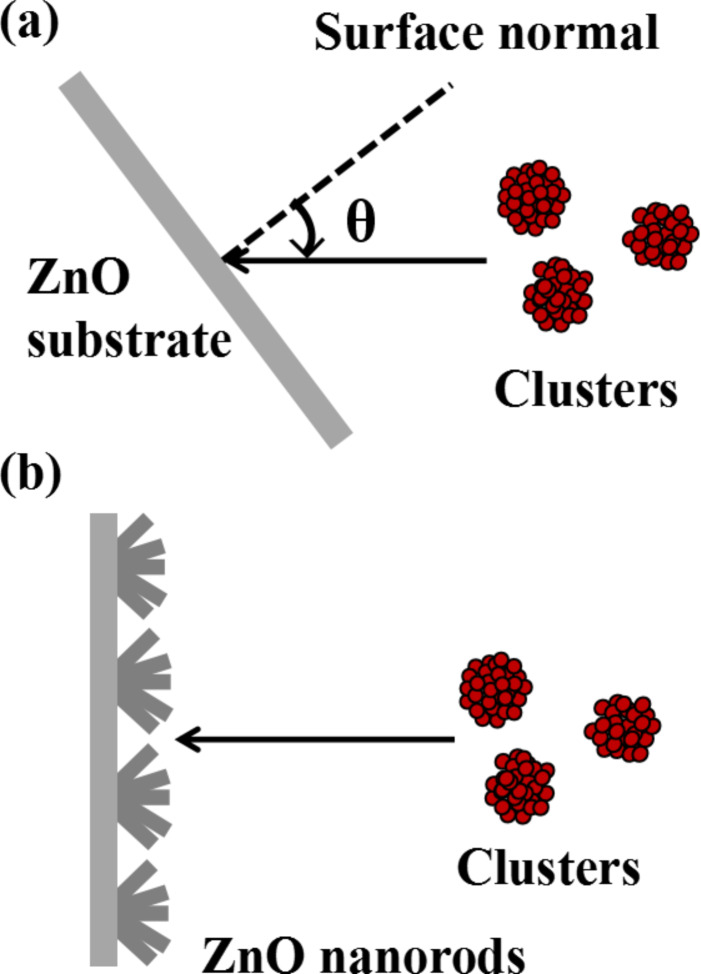
Schematic view of the irradiation experiments: (a) for ZnO flat substrates, θ is the variable incidence angle, and (b) for ZnO nanorods.

For cluster ion beam treatment we use a custom-built gas cluster accelerator described elsewhere [18]. The Si substrates with grown ZnO nanorods and ZnO substrates were cut into 2 × 2 mm samples and irradiated by Ar cluster ions with an average specific size of 1000 and accelerated by voltages in the range of 5–10 kV with a fluence in the range of 10^16^–10^17^ cm^−2^. For the flat single crystal ZnO substrates the incidence angle was varied in the range of 0–80° by rotating the sample holder. The incidence angle is defined as the angle between the normal of the sample surface and the ion beam direction. The experimental geometry is schematically shown in Figure 1a. The samples with ZnO nanorods were irradiated under an incidence angle of 0° (Figure 1b).

After the cluster bombardment, the modified surface morphology of the flat substrates and nanorods was studied with a scanning electron microscope (SEM) Zeiss Sigma, operated at 20 kV accelerating voltage. An atomic force microscope (AFM) Shimadzu SPM-9500J3 was used to study the ripple formation on the flat ZnO substrates. The AFM was operated in tapping mode with measuring areas of 2 × 2 and 5 × 5 µm.

## Results and Discussion

### Single crystal ZnO substrates

**Figure 2:**
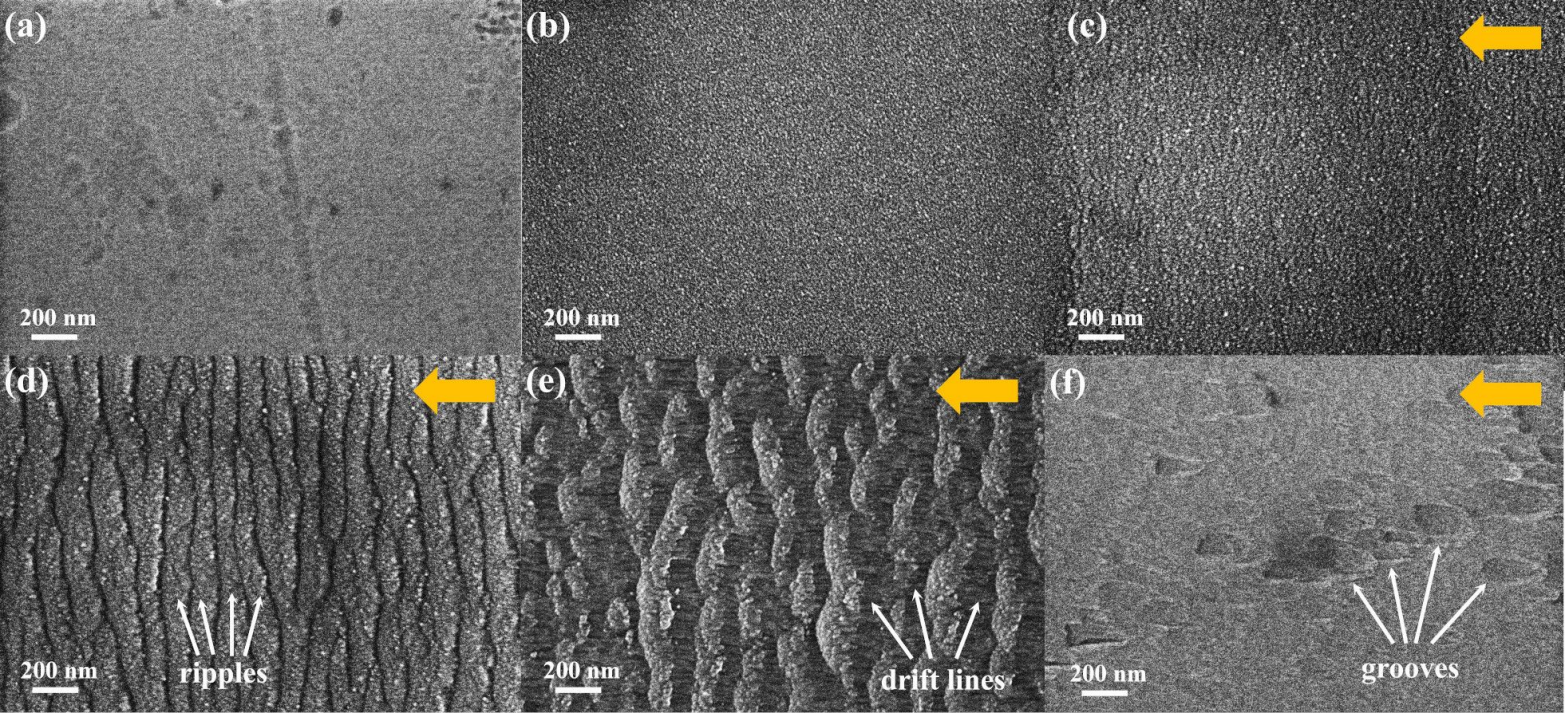
SEM images of ZnO substrate surfaces (a) before and after irradiation at 10 kV with a fluence of 4 × 10^16^ cm^−2^ and different incidence angles: (b) 0°; (c) 30°; (d) 45°; (e) 60°; (f) 80°. Direction of the projection of incident GCIB is indicated by yellow arrows.

**Figure 3:**
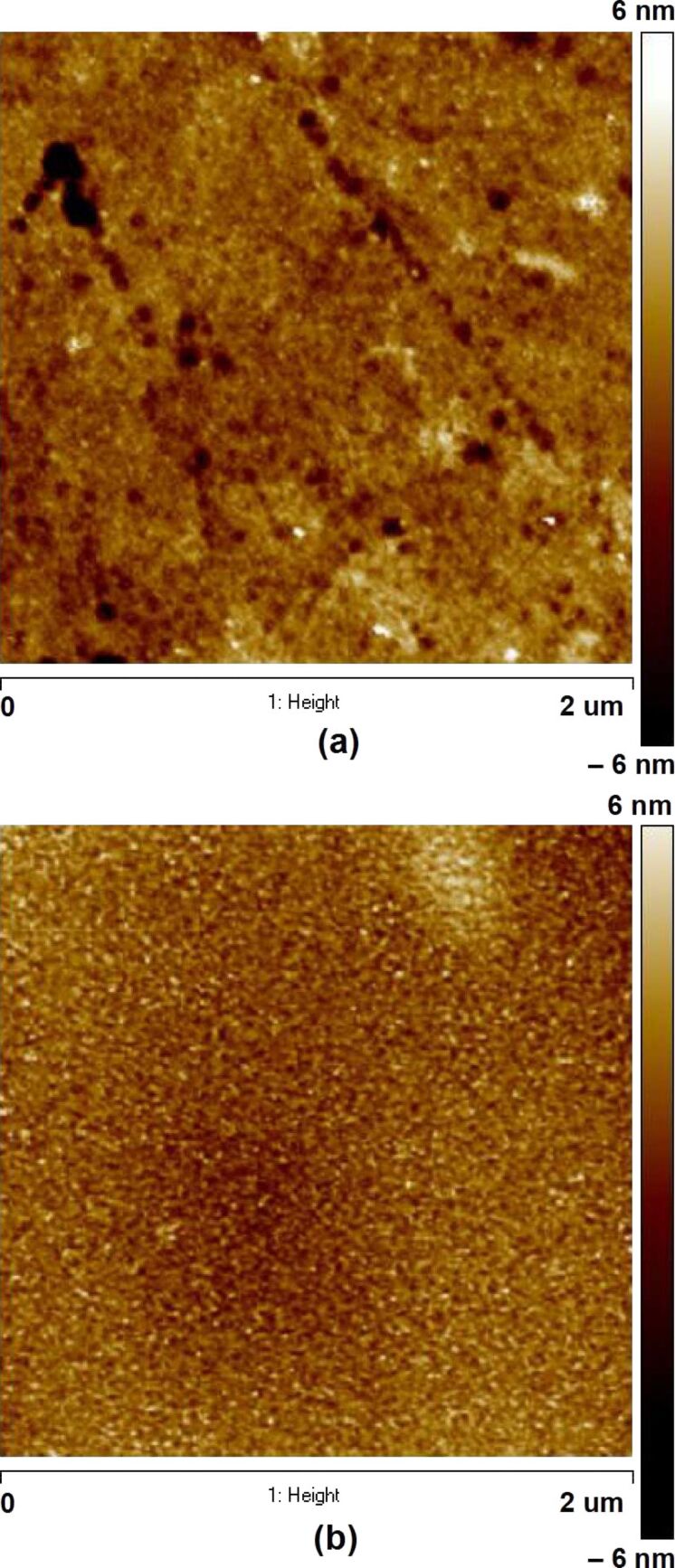
AFM images of ZnO substrate surfaces (a) before and (b) after normal irradiation at 10 kV and fluence of 4 × 10^16^ cm^−2^.

First, we studied the influence of GCIB irradiation on flat ZnO single crystal samples. Their large flat surface allows to determine the main dependencies of ripple formation on the GCIB parameters (incidence angle, accelerating voltage, and fluence). SEM and AFM images shown in Figure 2 and Figure 3, respectively, present the surface morphology of the substrates before and after Ar cluster bombardment at different incidence angles, θ = 0–80°. The acceleration voltage and ion fluence were 10 kV and 4 × 10^16^ clusters/cm^2^, respectively. Scratches and pits of 50–100 nm in size and 5 nm in depth are visible on the substrate surface before irradiation (Figure 2a and Figure 3a). After irradiation at θ = 0° the scratches and pits disappeared and features ca. 10 nm in size homogeneously dispersed all over the surface formed (Figure 2b and Figure 3b). These surface features can exhibit overlapped craters formed after collisions of the accelerated clusters with the surface. The surface roughness after normal cluster irradiation slightly decreases from initial 0.8 nm to 0.6 nm. After irradiation at θ = 30°, slightly blurred ripples perpendicular to the GCIB direction appear (Figure 2c). When the incidence angle increases up to 45°, these ripples become very evident (Figure 2d). At this angle two kinds of morphological features can be seen. First, there are light-gray ripples, which are composed of less dense material moved by ion-stimulated diffusion and, second, there are the dark-gray lines of the clean surface representing valleys between the ripples. A further increase of the incidence angle up to 60° results in the further development of the ripples and valleys, i.e., their wavelength increases (Figure 2e). Moreover, in the valleys fine drift lines parallel to the GCIB direction are observed, similar to those found on a gold surface [19]. The drift lines are parallel to the surface projection of the incidence ion-beam direction and perpendicular to the adjacent ripples. These drift lines are the paths formed by the target atoms in the course of mass redistribution processes forced by the continuous cluster impacts. At an incidence angle of 80°, the ripples, valleys, and drift lines disappear. Instead, large droplet-like grooves parallel to the incident direction are formed (Figure 2f). These grooves represent the pits and scratches on the initial surface modified by the cluster beam bombardment. The shape of the grooves is similar to those formed on a gold surface irradiated by the GCIB at 70° [19]. The absence of visible ripple morphology in this case can be explained by the smoothing effect, which is also observed at grazing incidence angles, when the cluster ion beam can effectively remove all surface irregularities [20]. Besides, at grazing incidence angles, due to a small transverse velocity the energy transfer from GCIB to the surface is reduced, which in turn decreases surface erosion and mass redistribution of the target atoms [19].

The most distinctly developed nanoripples are formed at 45° and 60° in agreement with the literature data for many other materials [11,21–22]. However, one can notice a notable difference between the morphology of the ripples and valleys formed at 45° and 60°. The ripples and valleys formed at 45° are rather well-ordered and the valleys remind of meandering streams, whereas the ripples formed at 60° become less ordered with reduced width, similar to aeolian sand ripples.

**Figure 4:**
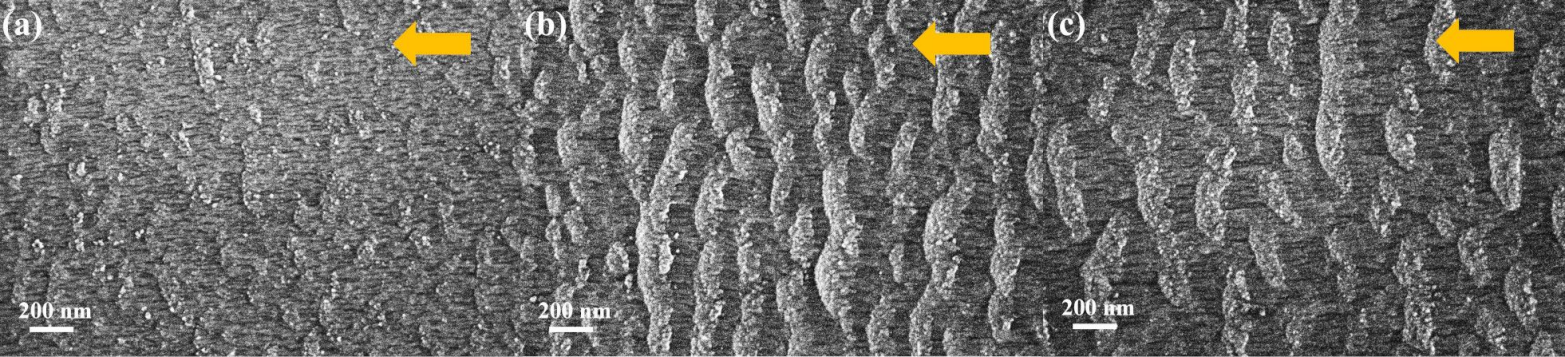
SEM images of ZnO substrate surfaces after irradiation at different ion fluences: (a) 10^16^ cm^−2^, (b) 4 × 10^16^ cm^−2^, and (c) 10^17^ cm^−2^ at an incidence angle of 60° and an accelerating voltage of 10 kV.

Next, we studied the dependence of the ripple formation on the ion fluence at an incidence angle of 60°, an accelerating voltage of 10 kV and a fluence in the range of 10^16^–10^17^ ions/cm^2^ (Figure 4). At the low fluence the drift lines prevail and the beginning of ripple formation can be observed. With increasing fluence, the crests of the ripples are developed and wavelength and height of the ripples calculated from the bottom drift line surface increase (Figure 4b and Table 1). This behavior is in agreement with data obtained for SiO_2_ films and gold surfaces bombarded with Ar clusters [19,22].

**Table 1:** The wavelength and height of the ripples formed on the flat ZnO substrates at different incidence angles, accelerating voltages and fluences measured by using AFM techniques.

incidence angle	acc. voltage	5 kV	10 kV	10 kV	10 kV
	ion fluence	4 × 10^16^ cm^−2^	10^16^ cm^−2^	4 × 10^16^ cm^−2^	10^17^ cm^−2^

45°	wavelength	—	—	141 ± 8 nm	—
	height			22 ± 3 nm	

60°	wavelength	176 ± 10 nm	134 ± 9 nm	237 ± 15 nm	290 ± 12 nm
	height	24 ± 3 nm	24 ± 2 nm	26 ± 3 nm	29 ± 2 nm

The process of ripple formation is triggered by the collisions of individual clusters with the surface. During the collision the cluster excavates the target material, which is deposited downstream nearby the impact spot. The accumulation of the excavated material during continuous irradiation gives rise to the formation of ripple [15] and the subsequent increase of the ripple wavelength and height (Table 1). However, the observed dependence of the ripple wavelength on the cluster fluence contradicts the BH theory, which predicts a descending dependence of the wavelength λ on the ion fluence *D*, i.e., λ ~ *D*^−1/2^ [9]. This result is to be expected, since the BH theory is based on the Sigmund linear collision theory, whereas the interaction of a cluster with the solid surface is a highly nonlinear process [13]. Here, it should also be noted that required fluence for ripple formation by cluster ion irradiation is usually more than one order of magnitude lower than that for monoatomic beams [1,8,15]. The fast ripple formation by cluster irradiation can be a result of the more effective sputtering (excavation) processes by cluster ions described above.

An influence of the accelerating voltage on the ripple formation is also revealed. Experiments have shown an increase of the ripple wavelength from 176 ± 10 nm to 237 ± 15 nm with increasing accelerating voltage at an incidence angle of 60° (Table 1). The dependence of the ripple formation on the accelerating voltage (energy) is not widely covered in the literature. The dependence on the cluster size at the same cluster energy for gold surfaces is known [11], which can be interpreted as an energy-per-atom dependence. In our study, the revealed energy dependence can be explained similarly to the fluence dependence. It is known that at higher cluster energy the sputtering yield of a target material increases [13]. If the amount of the material deposited downstream after a cluster collision is proportional to the sputtering yield, then one can conclude that the accumulation of the excavated material occurs faster at elevated cluster energy. As a result, the development of the ripple structure occurs faster at a higher cluster energy, which is observed as an increase of the ripple wavelength.

### ZnO nanorods

**Figure 5:**
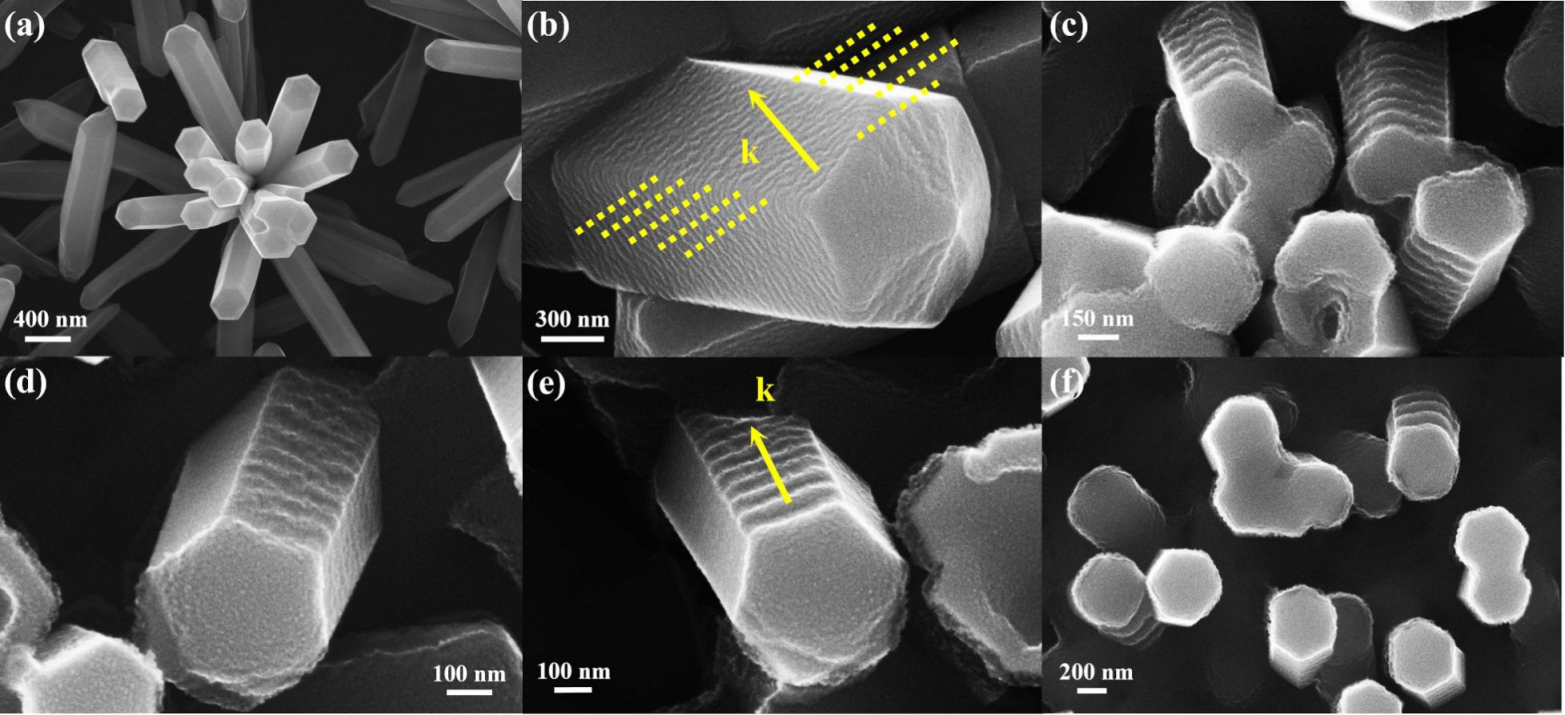
SEM images of modified ZnO nanorods at different cluster energies and fluences: (a) as grown nanorods, (b) 5 kV and 10^16^ cm^−2^, the yellow dotted lines are parallel to the ripples, the yellow arrow represents the direction of the wave vector *k*, (c) 5 kV and 4 × 10^16^ cm^−2^, (d) 10 kV and 10^16^ cm^−2^, (e) 10 kV and 2 × 10^16^ cm^−2^, and (f) 10 kV and 4 × 10^16^ cm^−2^.

At first, it should be noted that the ZnO nanorods have six facets and have been grown in clustered structures with different angles between each nanorod axis and the surface normal (Figure 5a). Therefore, for such material the effect of GCIB irradiation should depend on the local incidence angle. By other words, each nanorod and each of the facet exposed to the irradiation has its own ripple morphology with corresponding ripple wavelength.

Different angles between the nanorods and the surface normal as well as the different nanorod lengths complicate a precise estimation of the ripple wavelengths. Nanorods irradiated by 5 and 10 kV clusters with fluences in the range of 10^16^–10^17^ cm^−2^ and at incidence angles of 45–70° are shown in Figure 5b–f. The estimated wavelengths of nanoripples formed on nanorods bombarded at 5 kV are 37 ± 11, 61 ± 14, and 73 ± 20 nm for fluences of 10^16^, 2 × 10^16^, and 4 × 10^16^ cm^−2^, respectively, and for nanorods bombarded at 10 kV are 75 ± 19, 79 ± 20, and 151 ± 42 nm for the same fluences. Thus, dependencies of the ripple wavelength on the accelerating voltage and ion fluence are qualitatively the same as those of the flat targets. The obtained result can be compared with a study by Ghoniem et al. on the sputtering of Re and W nanorods with low-energy argon ions in which the authors have found the formation of rather weak ripple structures on the stem side of the nanorods with a wavelength of 300 nm [23].

The shortest wavelength is observed for 5 kV bombardment with the lowest fluence of 10^16^ cm^−2^ (Figure 5b). From the upper facet of a large nanorod one can see that the ripples are not perpendicular to the nanorod side edges (Figure 5b), i.e., the direction of the wave vector *k* of the ripples is not parallel to the nanorod axis. Hence, it can be concluded that in the case of low energy and fluence the ripple formation is controlled by the orientation of the ion incidence projection on the facet surface under irradiation, analogously to the flat substrates. As accelerating voltage and fluence increase (Figure 5c–f) a new effect can be observed. Now all ripples are perpendicular to the nanorod axes (the wave vector is parallel to the axes) independently of the orientation of ion incidence projection on the facet. Thus, the ripple formation is now controlled by the orientation of the nanorod side edges. A similar “guiding” effect was observed in experiments where the boundary of irradiated regions was used to template the lateral ripples formed by focus ion beam irradiation [24]. From these findings one can conclude that the “guiding” effect is observed if the surface under irradiation has boundaries or edges, i.e., is laterally confined, and if the ripple wavelength becomes comparable with the lateral size of this confined surface, compare Figure 5b and Figure 5e.

**Figure 6:**
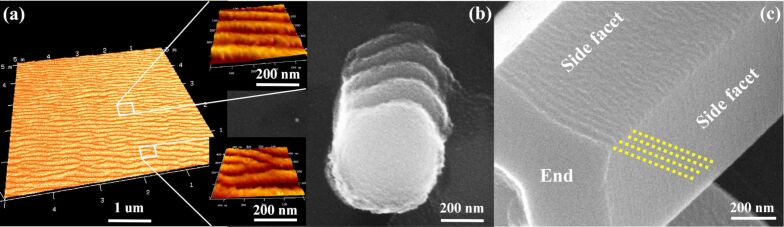
(a) AFM image of nanoripples formed on the ZnO substrate. Insets show images on a larger scale. (b) SEM image of ribs on the ZnO nanorod after irradiation at 10 kV Ar clusters with 4 × 10^16^ ions/cm^2^ fluence at 45° to the surface normal. (c) ZnO nanorod after irradiation at 5 kV and 10^16^ cm^−2^, the yellow dotted lines are parallel to the ripples formed near the end edge.

Comparing Figure 5d and Figure 5e one more effect can be observed. After low-fluence irradiation, the ripples are disordered and remind of aeolian sand ripples, similar to the those observed on the flat samples (see Figure 5d and Figure 4). Whereas, if the fluence is doubled ordered step-like ripples are formed (Figure 5e). At even greater fluence and appropriate incidence angles the ordered ripples are further developed. Some of the nanorods lose the hexagonal shape and resemble ribbed cylinders (Figure 5f). This ordering effect is not observed for the flat ZnO surface under any irradiation conditions. The ripple morphology on the flat ZnO surface and an example of a ripple “defect” are shown in Figure 6a and the lower inset of Figure 6a, respectively. Whereas, on the nanorod surfaces well-ordered and almost defectless ripples are formed (Figure 6b and Figure 5c,e,f). Similar to the “guiding” effect mentioned above, an ordering effect (at an appropriate fluence) is also observed if the ripple wavelength is comparable to the characteristic size of the area under irradiation, i.e., when such an area is laterally confined.

The role of surface lateral confinement in the ordering of the ripples can be discussed also from another point of view. Carter et al. have found that local defects of the flat surface (initially existing or created by the irradiation) initiate the formation of ripple trains [8,25]. If a nanorod is exposed to the irradiation, then the edges between the side facets and the ends can play the role of such surface defects. Our experiment does confirm the role of boundary surface defects in ripple formation. In Figure 6c one can see the formation of two to three ordered nanoripples oriented parallel to the end edge. A similar mechanism influences the ripple formation at higher energy and fluences, i.e., when the ripple wavelength becomes larger (Figure 5c,e,f). But this ripple formation mechanism gives no information about the large-scale ordering of the nanoripples observed on the nanorods at high ion fluences. If the object under irradiation is simply rescaled, then, in the case of the wavelength comparable to the nanorod facet size, only a few ripples formed near the end edge would be observed, similar to ripples in Figure 6c. However, Figure 5e clearly demonstrates that the number of ripples reaching seven to eight seems to be limited only by the length of the nanorod. Moreover, in our recent study a similar effect was observed for thin ZnO needles with a few tens of ripples [26]. Hence, a correlation between the ordering effect of the ripples and lateral confinement of the nanorod facet needs to be proposed.

The influence of the confinement on the ripple formation can be explained as follows. It is known that during crater formation the target material is sputtered in the azimuthal plane in all directions. Therefore, during normal cluster bombardment there is an isotropic flow of sputtered material parallel to the surface plane. If the incidence angle is not equal to 0°, the azimuthal distribution of the sputtered material becomes asymmetric with a prevalence of particles ejected in the downstream direction. At the same time, there are still particles ejected in the perpendicular directions [15]. Thus, in the case of a nanorod facet, the lack of a surface adjacent to the facet results in a lack of particles that could be sputtered from this surface and deposited on the given nanorod facet. This change in the flow balance of the sputtered particles should have an effect on the ripple formation, in particular, introduce a stabilizing factor to the formation of well-ordered ripple structures.

A recent theoretical study by Motta et al. predicting a remarkably defect-free ripple formation on the plane surface by ion bombardment of a binary material should also be noted [10]. In this theory, the composition change of the surface layer by the ion bombardment is discussed and a defect-free ripple formation of an elemental material becomes impossible from this point of view. In our experiment we also use binary materials and a defect-free ripple formation is observed, but only for nanorods. However, here, the scenario described in [10] seems unlikely due to a very low energy per atom in the cluster (a few electronvolts) and a consequently very low probability of any composition change of the material under irradiation.

## Conclusion

Using Ar^+^ cluster ion beam irradiation we have formed nanoripple array structures on ZnO single crystal substrates and facets of ZnO nanorods. The nanoripple formation is significantly governed by the cluster beam incidence angle, energy, and fluence. For the flat surface the ripple formation starts at an incidence angle of 30°. The most distinctly developed nanoripples are observed at incidence angles within the range of 45–60°. The nanoripple wavelength and height increase from 134 to 290 nm and from 24 to 29 nm, respectively, with increasing accelerating voltage and fluence. The formation of the ripples also occurs on the facets of nanorods. Similar wavelength dependencies on the accelerating voltage and fluence have been found for nanorods. However, in comparison with the flat surface, nanoripples on the nanorod facets at high irradiation fluences exhibit “guiding” and ordering effects. The former leads to the independence of ripple formation direction on the beam projection orientation, i.e., that orientation of the ripple wave vector is always parallel to the nanorod axis. The latter leads to the formation of ripples, which resemble parallel steps rather than aeolian ripples. It is suggested that the “guiding” effect can be attributed to the presence of the nanorod end edge, which plays initiates the ripple structure formation. The ordering effect can be connected with a change in the flow balance of the sputtered target material due to confinement of the nanorod facets in comparison to the “quasi-infinite” flat substrate.

In this study we formed nanoripples only on nanorod facets that were in the beam. In other words, only half of the nanorod side facets were modified. To obtain nanorods entirely covered with ripples with the same wavelength it is necessary to grow well-aligned vertical nanorods and rotate the sample around the nanorod axes during off-normal cluster bombardment.
